# Fungal contamination patterns and surface material susceptibility in manufacturing environments

**DOI:** 10.1093/sumbio/qvaf029

**Published:** 2025-12-03

**Authors:** Nixon Anthony, Wen Chun Chan, Che Hsien Huang, Jie Han Li, Hsin Hui Chiu, Li Ya Hung, Kuan Chieh Chen

**Affiliations:** Mold Research Center, YCM PRODUCTS CO., LTD., 509 Jinma road, Changhua 500, Taiwan; Mold Research Center, YCM PRODUCTS CO., LTD., 509 Jinma road, Changhua 500, Taiwan; Mold Research Center, YCM PRODUCTS CO., LTD., 509 Jinma road, Changhua 500, Taiwan; Mold Research Center, YCM PRODUCTS CO., LTD., 509 Jinma road, Changhua 500, Taiwan; Mold Research Center, YCM PRODUCTS CO., LTD., 509 Jinma road, Changhua 500, Taiwan; Mold Research Center, YCM PRODUCTS CO., LTD., 509 Jinma road, Changhua 500, Taiwan; Mold Research Center, YCM PRODUCTS CO., LTD., 509 Jinma road, Changhua 500, Taiwan

**Keywords:** alpha-diversity, *Aspergillus*, bioburden, biodeterioration, beta-diversity, zero-inflated negative binomial regression

## Abstract

Fungal contamination poses significant quality and health risks in manufacturing environments, yet patterns and environmental drivers in textile sectors such as footwear manufacturing remain poorly understood. This study examined surface bioburden and fungal diversity across materials in Indonesian footwear manufacturing facilities by combining culture-based enumeration with environmental monitoring. We collected 84 surface samples, 43 contaminated samples were sequenced, revealing 21 fungal genera. Material type had minimal effect on contamination (Kruskal–Wallis H = 9.342, *P* = 0.0531, *ε²* = 0.0008), though wood surfaces consistently showed higher loads. Humidity was the most consistent environmental predictor, while CO₂ correlated positively with wood contamination (*β* = 0.01, *P* = 0.004) but negatively with leather (*β* = −0.01, *P* = 0.01), suggesting surface-dependent operational influences. *Aspergillus* and *Penicillium* were the dominant genera, with no substrate-specific clustering. Zero-inflated negative binomial models more accurately captured contamination patterns on fabric and plastic, while standard models were more suitable for consistently contaminated materials. These results highlight humidity as a key predictor of surface contamination and suggest that environmental monitoring could inform contamination surveillance strategies in footwear manufacturing.

**Sustainability Statement**

This study contributes to SDG 12 (Responsible Consumption and Production) by establishing practical frameworks for manufacturing environments. Demonstrating that environmental parameters can predict surface contamination, this study provides a resource-efficient alternative to labor-intensive sampling protocols. The integrated biomonitoring approach enables proactive contamination management, reducing product losses and material waste from microbial deterioration in footwear and textile manufacturing. The finding that operational conditions exert stronger influence than material type emphasizes broad-based environmental management strategies. These findings support sustainable industrial practices by minimizing biodeterioration-related waste while maintaining product quality in high-humidity tropical manufacturing settings, directly addressing resource efficiency and waste reduction goals.

## Introduction

Microbiological monitoring is essential across pharmaceutical manufacturing, building maintenance, and cultural heritage preservation (Caggiano et al. 2014, Borrego et al. 2022, Marsit et al. 2025). Surface microbial loads can cause product deterioration, economic losses, and health risks (Souza et al. 2023, Hammad et al. 2025). While environmental control strategies have been widely studied in pharmaceutical and food industries (Konsa et al. 2022), systematic bioburden assessment in other manufacturing sectors remains limited. A recent study has also demonstrated the vulnerability of textiles to contamination during shipping (Hammad et al. 2025) as well as the presence of ascospores in ingredients, packaging materials, and processing environments within the food industry (Rico-Munoz 2017). Fungal spores in industries like dairies, greenhouses, carpentries, textiles, and pharmaceuticals not only pose serious health hazards but also contribute to increasing product recalls (Cosentino and Palmas 1991, Vijayakumar et al. 2016).

Environmental parameters such as temperature, humidity, and CO₂ levels play a significant role in fungal growth and dispersal. Elevated humidity promotes spore release, with fungal growth thriving in indoor environments where relative humidity (RH) ranges from 60% to 100%, and temperatures between 19°C and 24°C are common (Zhang et al. 2010, Loukou et al. 2024). Furthermore, elevated CO₂ levels, often associated with human occupancy and poor ventilation, can indirectly encourage fungal proliferation by signaling high activity zones where contamination is more likely (Chao et al. 2002). Particulate matter, specifically PM₁₀, is also crucial in assessing airborne fungal contamination, as fungal spores, which typically range from 2–10 *μ*m in diameter, contribute to the organic carbon load in indoor air and pose potential occupational health risks (Zhang et al. 2010, Kumar and Attri 2016). Surface contamination, on the other hand, arises from multiple sources, including human activity, settled dust, and incoming materials (Sandle 2015, Borrego et al. 2022). While these parameters are often studied in isolation under controlled conditions, the complex interactions between them in active manufacturing environments remain poorly understood, highlighting a significant gap in current research on fungal contamination and deterioration in such settings.

Textiles are particularly vulnerable to fungal contamination due to their porous structure and moisture sensitivity (Sanders et al. 2021). Fungal colonization can lead to discoloration, odor, and structural degradation, with global losses in textiles estimated at 920 000 tons annually (Mushtaq et al. 2025). In footwear manufacturing, where textiles are combined with materials such as leather, rubber, plastics, and wood, the distinct properties of each material create varied contamination risks (Muthu 2013). Fungal colonization on textiles results in visible damage such as discoloration, odor, and reduced tensile strength (Szostak-Kotowa 2004, Konsa et al. 2022). Leather, rich in proteins, lipids, and moisture, is particularly susceptible to fungal attack under humid conditions (Song et al. 2025). Similarly, rubber components in footwear, such as outsoles and insoles, are vulnerable to structural degradation and weight loss from microbial activity (Mollea and Bosco 2020, Krivushina et al. 2022).

Manufacturing equipment can act as a vector for cross-contamination (Aviat et al. 2016), while commonly used industrial materials such as wooden pallets, plastic molds, and synthetic covers provide surfaces for fungal spores to persist and spread. Though material properties influence microbial load, it is ultimately the intersection of hygiene practices and the environmental microclimate that determines contamination outcomes (Yang and Li 2007, Perez et al. 2024).

Textile materials offer a favorable substrate for fungal growth, due to the organic nutrients that support the proliferation of molds (Sanders et al. 2021). This risk is exacerbated during post-manufacture shipping and storage, particularly under tropical conditions where elevated temperature and humidity levels accelerate fungal development. Spores originating in manufacturing facilities can germinate during transit, leading to hyphal growth and subsequent product deterioration. Such microbial degradation results in diminished material quality, increased business costs, reputational damage, and potential health risks to workers and consumers (Hammad et al. 2025).

Indonesia ranks among the world’s leading footwear exporters (APICCAPS 2025), making contamination control economically significant. In 2019, global footwear reached 21.2 billion pairs, with Asia largest producer (87.4%) and exporter (83.9%) in 2020 (Nurkomariyah and Sutjiatmo 2023) In 2023, the sector generated $6.5 billion in export revenue and employed over 819,000 workers in 2018 (Indonesia Ministry of Industry 2019, Hartoyo and Daspar 2025). With production concentrated primarily in Java (Goni and Kadarusman 2015), the industry operates within a high-risk tropical climate where warm temperatures and elevated humidity create optimal conditions for microbial contamination. Despite this, systematic assessments of fungal presence and its environmental determinants in industrial footwear facilities remain scarce.

This study developed practical surface contamination assessment protocols for tropical manufacturing environments, integrating culture-based enumeration with real-time environmental monitoring. We evaluated contamination patterns across materials, identified environmental parameters that predict microbial loads, and characterized the preliminary fungal diversity relevant to contamination control.

## Materials and methods

### Location introduction and sampling

Environmental and surface samples were collected from three industrial sites in Indonesia during the 2024/25 rainy season (BMKG 2024): Location C in Central Java (12 buildings; 11 November 2024) and Locations A and B in East Java (three buildings each; 2–3 January 2025). Indoor environmental parameters were recorded at each session, including air temperature (°C), relative humidity (RH, %), carbon dioxide (CO₂, ppm), and particulate matter (PM₁₀, *μ*g/m³), using a calibrated Temtop M2000 air quality monitor following Öztürk et al. (2024).

Surface types sampled across the three sites included fabric (*n* = 19), leather (*n* = 24), wood (*n* = 22), plastic (*n* = 10), and rubber (*n* = 9) with total of 84 samples. Sample sizes reflect the relative availability of each surface type within the manufacturing environments, with leather, fabric, and wood being more prevalent and accessible across production areas than plastic and rubber components. A full list of sampled surfaces is provided in Table S1. Sampling followed ISO 18593:2018 using sterile cotton swabs (Onemed) applied over 10 cm² per sample (ISO, 2018). Swabs were immediately placed in sterile Nasco Whirl-Pak^®^ bags and transported at ambient temperature in sealed dry microbiology bags without transport medium. Swabs were transported under ambient conditions and delivered to the laboratory within 48–72 h of collection, and were immediately cultured upon arrival. Standardized handling across all samples ensured consistency across sites and surface types. Ten sterile swabs were included as negative controls and were handled identically to assess potential contamination during sampling and transport.

Each swab was suspended in 1 ml of 0.005% Tween 80 solution and vortexed. The suspension was directly inoculated, without dilution, onto triplicate Potato Dextrose Agar (PDA; Thermo Fisher Oxoid™) plates supplemented with chloramphenicol (100 mg/l) (Microbiology Society, 2016). Plates were sealed with Parafilm™, incubated at 25°C–30°C and 75% RH for 3–5 days, and visually inspected for fungal growth. A growth kinetics experiment was also conducted with five surfaces to validate the 3 days incubation count methods on Fig. S2. Three-day incubation was considered adequate for estimating colony counts under operational time constraints (Sandle 2013, Black 2020). Mean colony counts across triplicates were used as representative values to minimize technical error. Representative fungal colonies were isolated and reincubated for 5 days under the same conditions to generate sufficient biomass for downstream sequencing.

### DNA extraction, sequencing

Representative colonies from the 51 contaminated samples were isolated and reincubated for 5 days under the same conditions to generate sufficient biomass for DNA extraction. Eight samples with mixed or agglomerated colony morphologies unsuitable for reliable isolation were excluded. DNA extraction from the remaining 43 samples was performed using the Quick-DNA™ Fungal/Bacterial Miniprep Kit (Zymo Research) with bead-beating mechanical lysis. DNA concentration and purity were assessed using a NanoDrop One spectrophotometer (Thermo Fisher Scientific). The ITS region was amplified using primers ITS5 and ITS4 in a 25 μl PCR reaction containing KAPA Taq polymerase. The cycling conditions were as follows: an initial denaturation at 95°C for 5 min; 32 cycles of denaturation at 95°C for 30 seconds, annealing at 55°C for 30 seconds, and extension at 72°C for 2 min; followed by a final extension at 72°C for 5 min. PCR products were subjected to Sanger sequencing using an ABI 3730XL DNA Analyzer (Applied Biosystems) with BigDye Terminator v3.1 chemistry.

Raw chromatograms were assessed for quality using Chromas, and consensus sequences were generated and aligned using ClustalW implemented in MEGA v12. Species identification was performed via BLASTn searches against the NCBI GenBank database with a similarity threshold of ≥ 98% (Table S2). The 43 sequenced samples yielded 40 unique species across 21 genera. For phylogenetic analysis, reference sequences from GenBank were aligned with study sequences using ClustalW. A maximum likelihood tree was constructed under the general time reversible (GTR) model with 1000 bootstrap replicates to visualize taxonomic relationships among identified genera.

### Statistical analysis

All statistical analyses were conducted in R (version 4.0.0), utilizing established packages for microbiome and ecological data. Because the environmental data were highly skewed and zero-inflated, nonparametric methods were employed. Normality was first assessed with the Shapiro–Wilk test. When assumptions of normality were met (*P* > 0.05), data were analyzed using ANOVA. For non-normal data, the Kruskal–Wallis test was applied. H statistics were reported for omnibus nonparametric tests. To quantify pairwise contrasts between surface types, Cliff’s *δ* was calculated as a nonparametric effect size, with 95% confidence intervals (CIs) estimated to assess precision and evaluate whether observed differences could be attributed to random variation.

Associations between environmental parameters and fungal contamination were modeled for each surface type using both Negative Binomial Linear Models (NBLM) and Zero-Inflated Negative Binomial Linear Models (ZINBLM). These models were selected for their suitability to overdispersed count data with excess zeros, common in environmental microbiological datasets (Zhang et al. 2017). Model fitting was implemented with the glmmTMB and pscl packages, widely applied in ecological and microbial research (Zeileis et al. 2008, Brooks et al. 2017). Model selection followed a forward stepwise procedure guided by Akaike Information Criterion (AIC). For regression models, Incidence Rate Ratios (IRR) with 95% CI were reported to facilitate interpretation of associations between predictors and contamination counts.

Fungal relative abundance was calculated as the proportion of colony-forming units (CFUs) for each genus relative to the total CFUs across all samples. Alpha-diversity metrics including observed richness, Chao1 estimator, Shannon index, and Simpson index were computed using the vegan package (Oksanen et al., 2025). The Kruskal–Wallis test was applied to test for significant differences in diversity indices among sample groups. For beta-diversity, samples containing only a single genus were excluded, as ordination requires multi-genus communities, reducing the dataset from 43 to 24 samples. Jaccard (presence/absence) and Bray–Curtis (abundance-weighted) dissimilarities were calculated. PERMANOVA via ADONIS tested for significant clustering by surface type.

## Results

### Environmental parameters of the sampling locations

**Figure 1.**
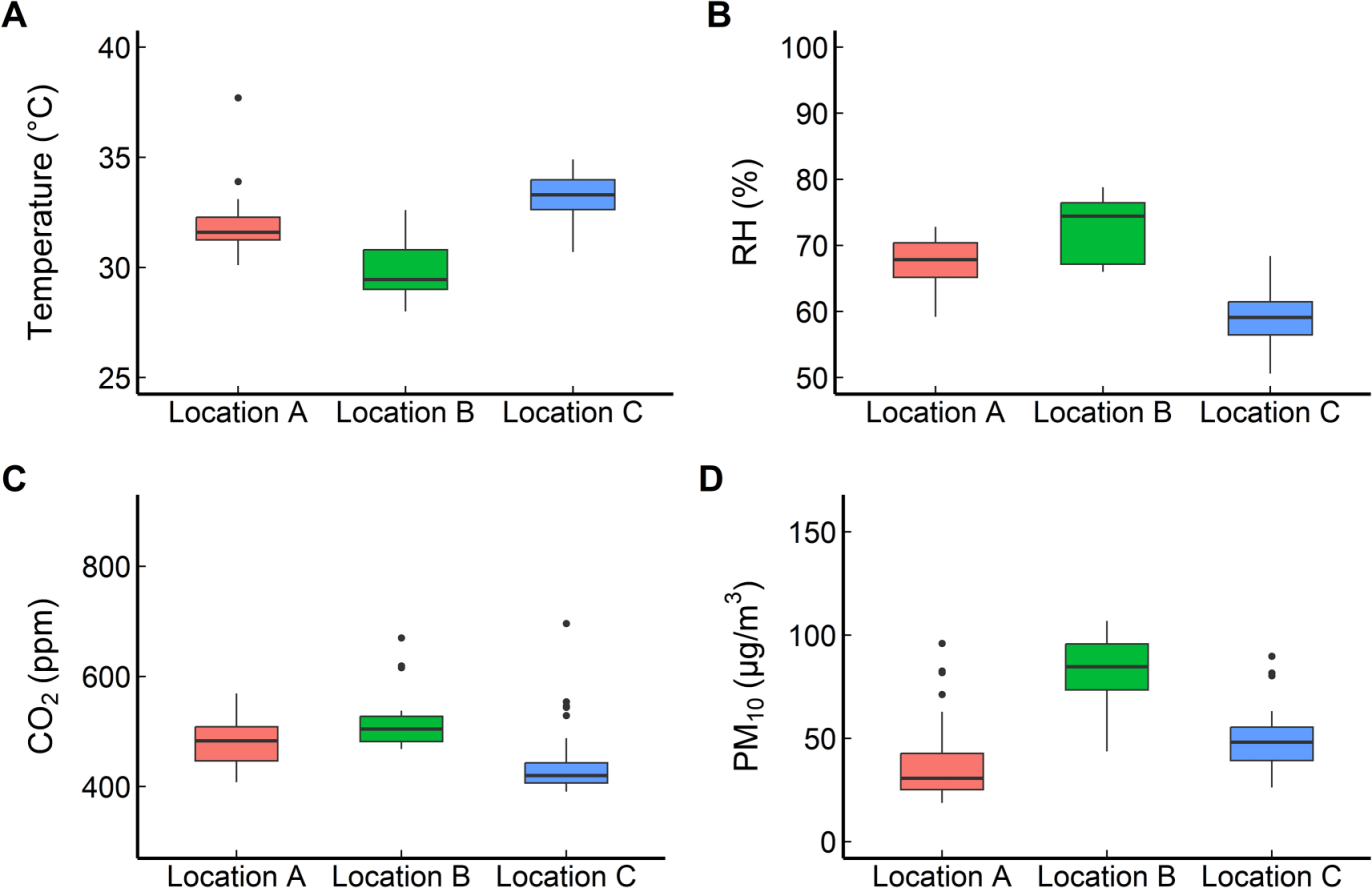
Boxplots comparing four key indoor environmental parameters across three sampling locations: (a) temperature (°C), **(b)** relative humidity (RH, %), (c) carbon dioxide (CO₂, ppm), and (d) particulate matter (PM₁₀, µg/m³). For (a) and (b), normality was confirmed (Shapiro–Wilk test *P* > 0.05), and differences were tested using ANOVA (*P* < 0.05). For (c) and (d), non-normality was detected (Shapiro-Wilk test *P* < 0.05), Kruskal–Wallis tests were used (*P* < 0.05).

Significant differences in indoor environmental conditions were observed across the three locations, as illustrated by the boxplots (Fig. 1). Location C recorded the highest temperatures (x̄ = 33.30°C, IQR: 32.65–33.98), while Location B exhibited the highest RH (x̄ = 74.45%, IQR: 67.18–76.43), PM₁₀ concentrations (x̄ = 84.70 µg/m³, IQR: 73.48–95.73), and elevated CO₂ levels (x̄ = 504.5 ppm, IQR: 481.75–527.50). Each location displayed distinct environmental characteristics, suggesting differences in ventilation systems or indoor air quality management practices. Despite all sampling being conducted during the monsoon season, the observed variations between locations indicated that indoor environmental conditions were primarily influenced by location-specific practices, building characteristics, and environmental controls rather than external meteorological factors.

### Surface contamination

**Figure 2.**
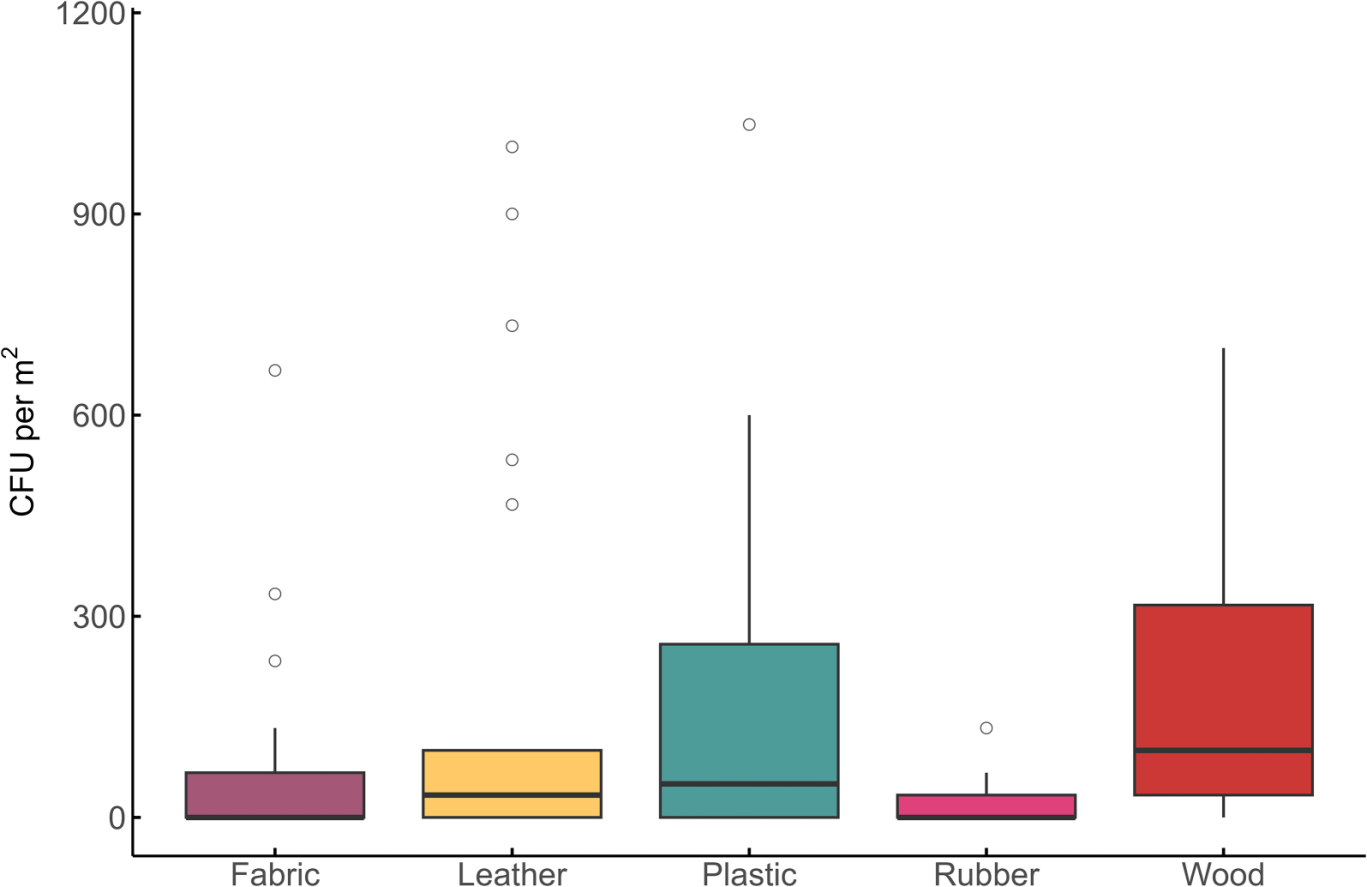
Boxplots of median for surface contamination levels (CFU/m²) across different material types. Points represent medians, and error bars indicate interquartile ranges (IQR). Kruskal–Wallis test: H = 9.342, *P* = 0.0531; effect size *ε²* = 0.0008.

Microbiological surface sampling revealed substantial variation across five surface types (Fig. 2). Due to extensive zero-inflation and tied ranks, nonparametric analyses were employed. The Kruskal–Wallis test showed marginally significant differences across surface types (H = 9.342, *P* = 0.0531, *ε²* = 0.0008). The very low effect size suggested minimal overall differences in contamination levels between surface types, likely due to high within-group variability from zero-inflation. The marginal significance combined with low overall effect sizes suggested that surface differences, while present, were small relative to the broader variability of contamination patterns in this industrial environment. The effect size findings aligned with the observed medians (Fig. 2). Wood (100 CFU/m²) and plastic (50 CFU/m²) showed the highest central tendencies, fabric and rubber medians were zero, and leather showed intermediate contamination (33.3 CFU/m²). Overall, these results indicated that wood surfaces are more prone to microbial accumulation compared to other materials.

**Figure 3.**
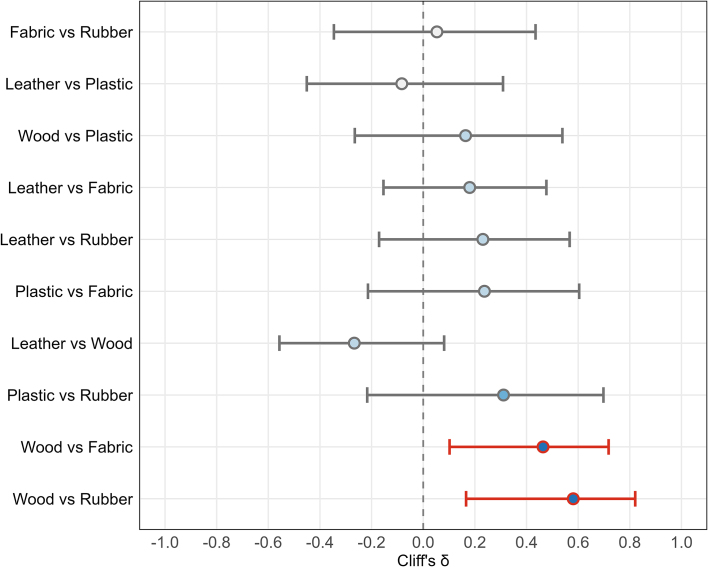
Pairwise effect sizes (Cliff’s *δ*) with 95% CIs for surface contamination comparisons. Colors represent effect size magnitude: ° = negligible (<0.147), 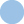
= small (0.147–0.33), 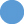 = medium (0.33–0.47), and 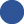 = large (>0.47). Red markers and intervals highlight statistically significant differences where CIs do not include zero.

The prevalence of zeros in the dataset (Table 1) likely contributed to reduced statistical power, making it difficult to distinguish subtle differences between surface types. Nonetheless, pairwise effect size analyses using Cliff’s *δ* (Fig. 3) revealed clearer contrasts. Wood consistently exhibited higher contamination than other materials, with large effects compared to rubber (*δ* = 0.581, 95% CI [0.166, 0.821]) and medium-to-large effects compared to fabric (*δ* = 0.464, 95% CI [0.102, 0.718]). Although the CIs remained wide from the zero inflation, these results indicated that certain material-specific patterns may still emerge despite the overall variability.

**Table 1.** Descriptive statistics across five surfaces.

	Sample size	Zero (%)	Median	Mean (CFU/m^2^)	SD	Max
Fabric	19	57.9	0	82.5	168.3	667
Leather	24	41.7	33.3	175	306.3	1000
Plastic	10	40	50	217	345.1	1033
Rubber	9	55.6	0	29.6	45.5	133
Wood	22	13.6	100	192	228.6	700

### Effects of environmental parameters on fungal contaminations on surfaces

Comparisons of model fit revealed mixed patterns across surface types. For leather, wood, and rubber, the negative binomial model (NBLM) outperformed the zero-inflated version (ZINBLM), producing lower AIC values and more stable estimates. In contrast, fabric and plastic were better described by ZINBLM (Table 2). It is important to note that AIC provides a measure of relative model fit rather than validation of individual predictors. Models that converged but yielded higher AIC were considered unnecessarily complex, while nonconverging models were excluded. Final model selection was therefore guided by the lowest AIC in combination with interpretability, avoiding added complexity where not required.

**Table 2.** Effect size between NBLM and ZINBLM. IRR and CI (95% CI).

	ZINBLM				NBLM			
Surface	AIC	Parameter	IRR	95% CI	AIC	Parameter	IRR	95% CI
Fabric	68.41	RH	1.04	[0.86; 1.26]	68.57	RH	1.39^+^	[0.99; 1.95]
		CO_2_	0.97**	[0.94; 0.99]		CO_2_	0.95	[0.91; 0.99]
		RH (logit)	0.68^+^	[0.43; 1.07]				
		CO_2_ (logit)	1.01	[0.93; 1.09]				
Leather	123.17	CO_2_	0.99*	[0.97; 0.99]	118.92	Temp	1.47	[0.93; 2.32]
		CO_2_ (logit)	0.99	[0.95; 1.03]		RH	1.18^+^	[0.99; 1.40]
						CO_2_	0.98**	[0.97; 0.99]
						PM_10_	1.04*	[1.00; 1.08]
Plastic	49.72	Temp	11.62***	[3.73; 36.23]	55.07	Temp	5.14	[0.62; 42.53]
		RH	2.06***	[1.54; 2.76]		RH	1.59*	[1.02; 2.47]
		CO_2_	0.99**	[0.98; 0.99]				
		Temp (logit)	2.98	[0.01; 1553.34]				
		RH (logit)	1.53	[0.28; 8.22]				
		CO_2_ (logit)	0.99	[0.92; 1.06]				
Rubber	29.68	CO_2_	0.99^+^	[0.97; 1.00]	21.73	RH	1.57	[0.73; 3.38]
		CO_2_ (logit)	0.99	[0.94; 1.04]		CO_2_	0.92	[0.81; 1.04]
Wood	124.45	RH	1.08*	[1.02; 1.15]	119.89	Temp	0.60**	[0.44; 0.83]
		CO_2_	1.01**	[1.01; 1.02]		CO_2_	1.01**	[1.01; 1.02]
		PM_10_	1.00	[0.98; 1.02]		PM_10_	0.98*	[0.96; 0.99]
		RH (logit)	0.12	[6.73E-07; 2.10E^+^04]				
		CO_2_ (logit)	0.72	[0.10; 5.02]				
		PM_10_ (logit)	2.72	[0.05; 156.78]				

Temperature (Temp), relative humidity (RH), carbon dioxide (CO_2_), and particulate matter (PM_10_).

+ = *P*-value < 0.1.

* = *P* -value < 0.05.

** = *P* -value < 0.01.

*** = *P* -value < 0.001.

For fabric, the difference in AIC between NBLM and ZINBLM was small when the same predictors were used. However, ZINBLM provided additional interpretive value: the RH logit term showed marginal significance [IRR = 0.68, 95% CI: 0.43–1.07, *p* < 0.1], indicating that under zero-inflated conditions, higher humidity decreased the probability of remaining in a structural zero state.

PM₁₀ effects were inconsistent across surfaces, with negative associations observed for wood (*β* = –0.01, *P* < 0.01) and positive associations for leather (*β* = 0.04, *P* < 0.01) in Table 3 . These opposing effects might have reflected the highly variable nature of PM₁₀, which fluctuated more sharply than other physical parameters, making it more sensitive to environmental disturbances and resulting in inconsistent contributions to model fit (Shakoor et al. 2020).

**Table 3.** Effects of environmental parameters on fungal contaminations on surfaces using ZINBLM and NBLM models.

	Count model			Logit model			Negative binomial model		
Surface	Parameters	*β*	*Z*	Parameters	*β*	*Z*	Parameters	*β*	*Z*
Fabric	Intercept	14.38**	5.00	Intercept	21.60	14.32	Intercept	2.81	6.10
	RH	0.04	0.10	RH	-0.39^+^	0.23	RH	0.33^+^	0.17
	CO₂	-0.04**	0.01	CO₂	0.01	0.04	CO₂	-0.05*	0.03
	Log(theta)	1.17	1.20						
Leather	Intercept	8.17*	3.30	(Intercept)	3.45	10.14	Intercept	-13.97	11.85
	CO₂	-0.01*	0.01	CO₂	-0.01	0.02	Temp	0.38	0.23
	Log(theta)	-0.85	0.64				RH	0.16^+^	0.09
							CO₂	-0.02**	0.01
							_PM10_	0.04*	0.02
Plastic	Intercept	-117.10***	27.09	(Intercept)	-57.57	148.26	Intercept	-80.80	48.60
	Temp	2.45***	0.58	Temp	1.09	3.19	Temp	1.64	1.08
	RH	0.72***	0.15	RH	0.43	0.86	RH	0.46*	0.23
	CO₂	-0.01**	0.01	CO₂	-0.01	0.04			
	Log(theta)	14.57	261.50						
Rubber	Intercept	6.76*	3.37	Intercept	2.61	10.77	Intercept	8.16^+^	4.41
	CO₂	-0.02^+^	0.01	CO₂	-0.01	0.03	RH	0.45	0.39
	Log(theta)	9.41	108.79				CO₂	-0.08	0.06
Wood	Intercept	-7.60**	2.62	(Intercept)	202.99	398.97	Intercept	14.86*	5.82
	RH	0.08*	0.03	RH	-2.13	6.16	Temp	-0.52**	0.16
	CO₂	0.01**	0.01	CO₂	-0.33	0.99	CO₂	0.01**	0.01
	PM_10_	-0.01	0.01	PM_10_	1.00	2.07	PM_10_	-0.01*	0.01
	Log(theta)	0.63	0.42						

Temperature (Temp), relative humidity (RH), carbon dioxide (CO_2_), and particulate matter (PM_10_).

+ = *P*-value < 0.1.

* = *P* -value < 0.05.

** = *P* -value < 0.01.

*** = *P* -value < 0.001.

For plastic surfaces, RH consistently emerged as a significant predictor across both models, whereas temperature estimates varied widely on. The ZINBLM of plastic suggested a strong positive association with temperature, but CIs were broad, reflecting both low sample size and the limited number of zero counts. This highlighted the challenge of applying zero-inflated models when excess zeros were sparse which NBLM yielded more reliable estimates on some surfaces. A similar pattern was observed for wood, where few zero counts led ZINBLM to produce unstable estimates and inflated AIC values, again favoring NBLM for stability.

Overall, both NBLM and ZINBLM provided useful insights for biomonitoring assessments in manufacturing environments. While NBLM generally offered more robust and stable estimates, ZINBLM was informative for surfaces with high proportion zero.

### Species diversity and surfaces

**Figure 4.**
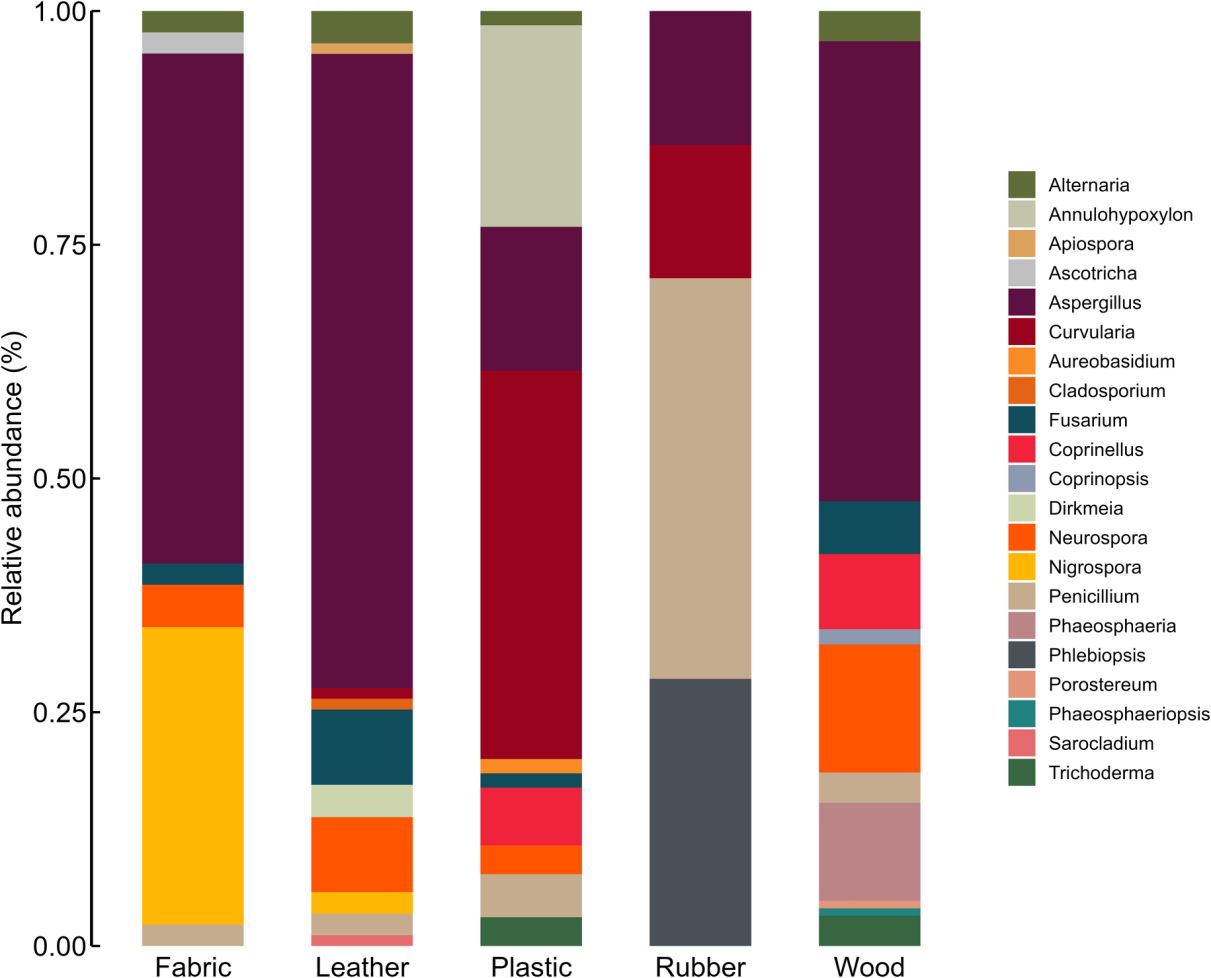
Relative abundance of fungal genera by surface type. Proportions based on CFU counts from 43 sequenced samples representing 21 genera.

Molecular identification of 43 samples revealed 21 fungal genera with *Aspergillus, Curvularia*, and *Penicillium* most frequently detected (Fig. 4). Alpha-diversity analyses showed no significant differences among substrate types (Table 4). Across substrates, Simpson index values were low (0.13–0.30), indicating strong dominance by one or a few genera. Chao1 also reflected low richness (1.71–3.11), with leather showing the highest estimated richness (3.11) and fabric the lowest (1.71). The similarity between observed richness and Chao1 suggested that sampling adequately captured the culturable diversity under the conditions employed. These results indicated that community structures were uneven and dominated by a few taxa, particularly *Aspergillus*.

**Table 4.** Alpha-diversity metrics of fungal communities across surface materials. Values are based on contaminated samples only: plastic (*n* = 7), wood (*n* = 18), fabric (*n* = 7), leather (*n* = 12), and rubber (*n* = 3).

	Genera detected	Shannon (*P* = 0.66)	Simpson (*P* = 0.63)	Chao1 (*P* = 0.40)
Fabric	7	0.26	0.15	1.71
Leather	11	0.51	0.29	3.11
Plastic	10	0.46	0.30	2.29
Rubber	4	0.19	0.13	1.33
Wood	12	0.44	0.26	2.31

**Figure 5.**
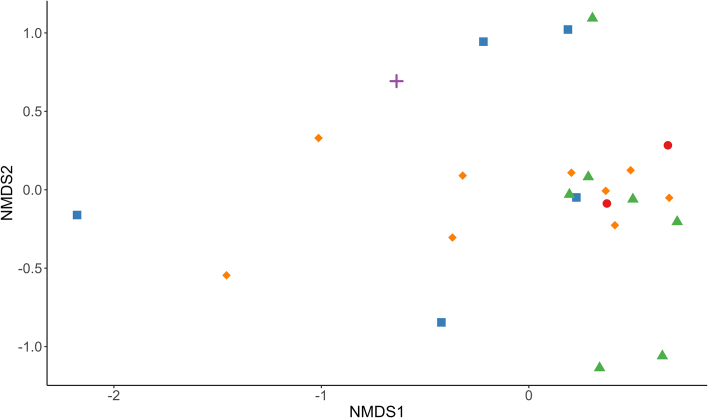
NMDS plot based on Bray–Curtis dissimilarity showing fungal community structure across samples. ADONIS test indicates no significant clustering by surface type (*p* = 0.34). The stress value of 0.12 suggests a good representation of the data in two dimensions. 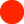 = Fabric, 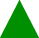 = Leather, 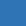 = Plastic, + = Rubber, 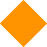 = Wood.

**Figure 6.**
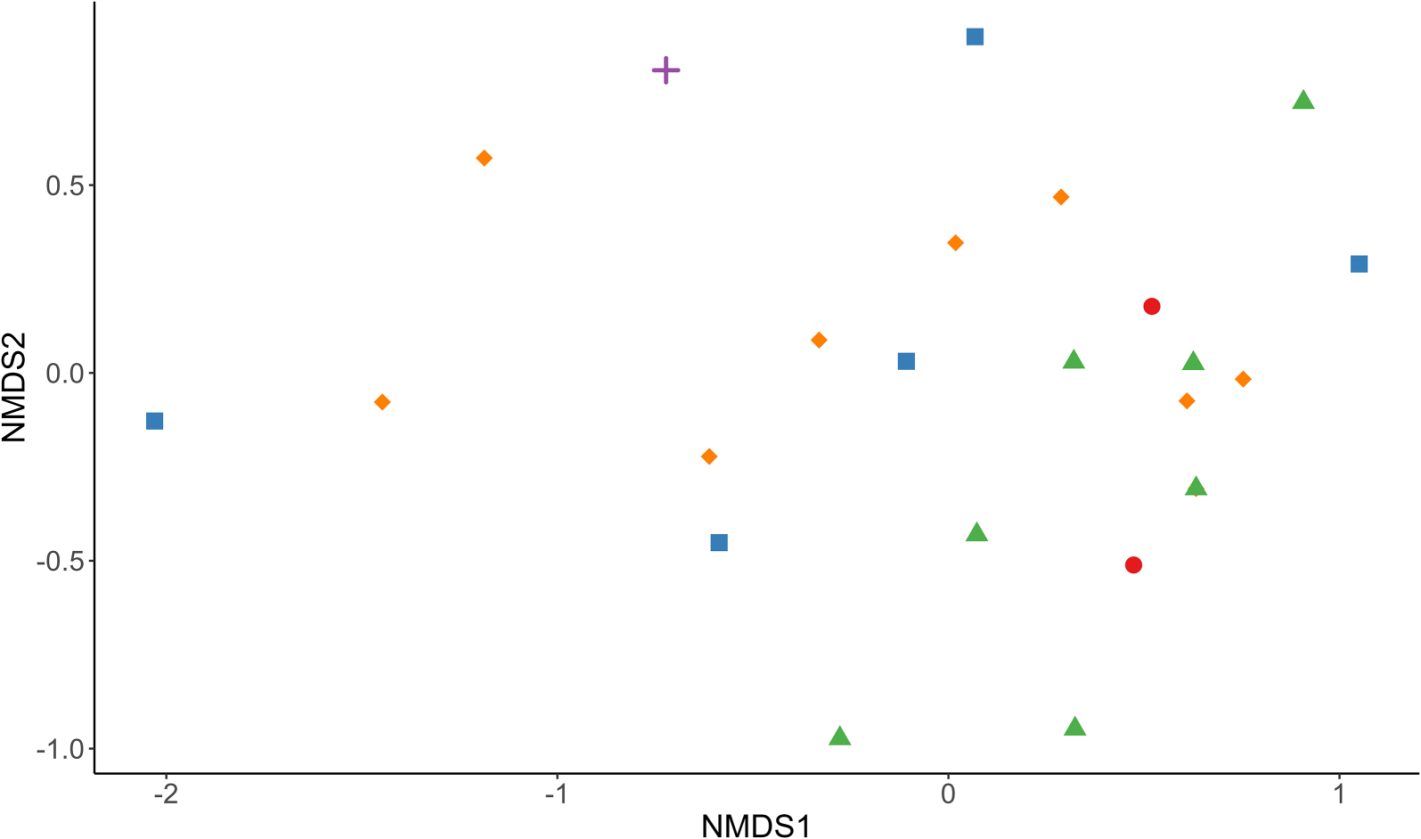
NMDS plot based on Jaccard dissimilarity showing fungal community structure across samples. ADONIS test indicates no significant clustering by surface type (*P* = 0.36). The stress value of 0.12 suggests a good representation of the data in two dimensions. 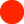 = Fabric, 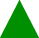 = Leather, 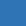 = Plastic, + = Rubber, 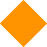 = Wood.

Beta-diversity analysis using nonmetric multidimensional scaling (NMDS) revealed no significant surface-specific clustering, with low stress values indicating reliable ordination on Jaccard (stress = 0.12, PERMANOVA *P* = 0.357) and Bray–Curtis (stress = 0.12, PERMANOVA *P* = 0.341) (Figs 5 and 6). The absence of distinct surface-associated communities indicates that fungal composition was similar across material types. The low diversity and dominance by *Aspergillus* and *Penicillium* are consistent with the selective recovery characteristics of PDA medium for fast-growing, culturable fungi.

### Phylogenetic tree

**Figure 7.**
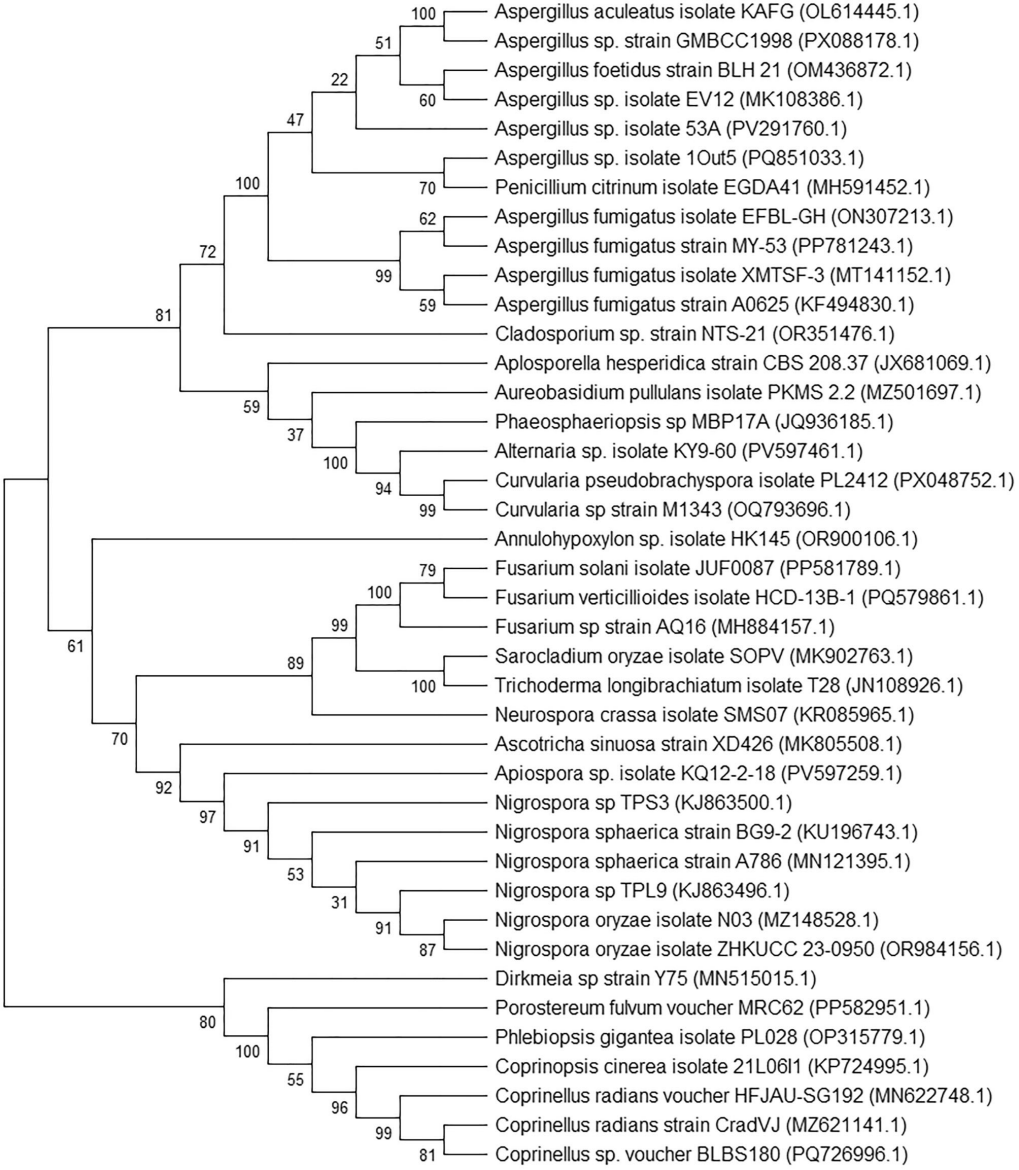
Phylogenetic tree constructed using the maximum likelihood method based on the General Time Reversible (GTR) model, inferred from ITS2 region sequences of fungal taxa identified from Table S2.

Phylogenetic analysis of cultured fungal isolates obtained from a manufacturer in Indonesia (Fig. 7) revealed a broad taxonomic spectrum, spanning the two major fungal phyla: *Ascomycota* and *Basidiomycota*. Most isolates belonged to *Ascomycota*, with *Aspergillus, Penicillium, Cladosporium*, and *Alternaria* emerging as the dominant genera. These genera were well-known for their capacity to colonize a wide range of materials, including dust, textiles, and building surfaces, and were sourced from both indoor environments (Sadrizadeh et al. 2022). Additional Ascomycete genera were identified, including *Curvularia, Nigrospora, Trichoderma*, and *Acremonium*, all previously reported in indoor air and settled dust in tropical and subtropical regions (Chao et al. 2002, Pitkäranta et al. 2011, Wang et al. 2017).

Four Basidiomycete genera (*Phlebiopsis, Coprinellus, Dirikmeia, Porostereum*) formed distinct, well-supported monophyletic clades. The detection of these wood-decay and litter-decomposing taxa suggested occasional colonization via outdoor air exchange or exposure with decaying organic materials (Pitkäranta et al. 2011). However, their relatively low abundance reflected both their lower prevalence in indoor environments and culture media usage. Using additional media could have increased the number of taxa detected. The phylogenetic tree confirmed robust species-level identifications, with all isolates forming well-supported clades consistent with reference sequences. This molecular validation strengthens confidence in the taxonomic assignments and provides a foundation for understanding fungal community composition in this tropical manufacturing setting.

## Discussion

The significant environmental differences observed across locations, despite all sites being sampled during the same monsoon season, indicate that indoor conditions were primarily shaped by facility-specific factors rather than external atmospheric conditions. Variations in CO₂ and PM₁₀ levels likely reflect differences in ventilation system performance and air exchange rates across facilities. Our data exhibited high variance across samples, which is a common feature of environmental bioburden datasets. Many surfaces yielded undetectable or zero colony counts, especially shortly after cleaning or in tightly controlled spaces, producing excess zeros and skewed distributions (Zhang et al. 2017). Sandle (2013) noted that microbial monitoring data rarely conform to parametric assumptions because counts are non-normal and variances are heterogeneous. These zeros and low counts, which inflate SDs (Table 1) reflect ecological reality. While surface physical characteristics such as roughness and porosity can affect spore retention (Whitehead et al. 2021, Hammad et al. 2025), our results suggest their effect is limited compared with operational and environmental drivers (Fig. 2). This suggests that operational factors in active manufacturing environments may override intrinsic material differences.

Contamination in manufacturing settings can arise through multiple pathways. Dust plays a critical role in indoor environments by transporting and depositing fungal spores (Chawla et al. 2023). Dust samples reflect both airborne and surface microbial communities. Comparisons between indoor dust and outdoor air reveal similar species diversity profiles (Chao et al. 2002, Pitkäranta et al. 2011), indicating that outdoor sources contribute to indoor contamination. It should be noted that the method used to detect fungi greatly impacts diversity estimates. Culture-based methods, while useful for quantifying viable organisms, significantly underrepresent total fungal richness due to their selectivity (Pitkäranta et al. 2011), a pattern also observed in this study.

Human activity further disperses spores through movement, re-aerosolization, and redeposition onto surfaces (Adams et al. 2013, Borrego et al. 2022). In addition, incoming raw materials may introduce fungal propagules that spread across different surfaces during processing (Sandle 2015, Zhao et al. 2025). These combined pathways suggest that operational and environmental factors drive the accumulation and redistribution of microbial loads, diminishing the relative contribution of intrinsic surface properties.

Humidity emerged as the most consistent predictor of surface contamination across all materials in this study. The mechanisms underlying this association likely operate through multiple pathways. Elevated humidity has been shown to increase airborne spore concentrations through enhanced spore release from fungal colonies (Zhang et al. 2010), potentially increasing deposition rates onto surfaces. Once deposited, moisture enhances surface wettability, promoting spore adhesion and retention (Whitehead et al. 2021). Additionally, humid conditions favor spore germination and subsequent mycelial growth (Zhao et al. 2020). Although our study did not directly test these mechanisms, the consistent humidity-contamination association suggests moisture operates through multiple pathways, which are increasing airborne spore availability and enhancing surface receptivity to colonization.

CO₂ associations varied by surface type, being positive for wood but negative for fabric, leather, and rubber. CO₂ is often used as a proxy for human occupancy (Andualem et al. 2019), but its associations with surface contamination may reflect operational activities rather than occupancy itself. For example, positive CO₂ associations on wood surfaces could result from direct handling during periods of higher human activity, whereas negative associations on mobile materials such as fabric or rubber may arise from cleaning or movement during those periods. While speculative, these patterns highlight that surface contamination may be influenced by the interaction of human activity and surface-specific operational use.

*Aspergillus* was consistently detected across all substrates, reflecting both its environmental abundance and rapid colonization capacity. This genus also recognized as capable of degrading natural textile fibers (Sanders et al. 2021). *Penicillium* was also frequent, consistent with its role as a common indoor contaminant in commercial areas (Shammi et al. 2021). Along with *Cladosporium* and *Alternaria*, these genera are regularly reported in industrial, indoor, and cultural heritage environments (Niemeier et al. 2006, Borrego et al. 2022) and are known to degrade both natural and synthetic materials under favorable conditions (Kavkler et al. 2015, Hammad et al. 2025).

The absence of distinct fungal communities across different surface types indicates that contamination originates primarily from common environmental sources such as airborne dispersal, settled dust, and incoming materials, rather than being shaped by the surface material itself (Borrego et al. 2022). Consequently, contamination control strategies should emphasize environmental management approaches including moisture control, air filtration, and handling procedures, recognizing that the dominant fungi can colonize a wide range of substrate materials.

Culture-based enumeration assessed viable fungal populations most relevant to contamination risk, revealing dominant genera that readily colonize surfaces under industrial conditions. However, reliance on a single growth medium inherently constrains the diversity detected (Wu et al. 2000). This approach likely underrepresented slow-growing taxa, organisms with specialized nutritional demands, and metabolically inactive fungi, leading to a narrowed community profile compared with culture-independent approaches, which typically capture a broader spectrum of taxa (Pitkäranta et al. 2011). Extended transport times may have reduced recovery of more sensitive organisms, further favoring resilient, rapidly sporulating fungi. Additionally, unequal sample sizes across materials reduced statistical power for material-specific comparisons. These constraints indicate that the 21 genera identified represent the readily culturable fraction relevant to rapid bioburden assessment rather than complete environmental diversity.

In conclusion, environmental monitoring effectively predicted surface contamination patterns in tropical footwear manufacturing. Humidity emerged as the most consistent predictor across materials, while CO₂ associations suggested surface-specific contamination dynamics linked to operational practices. Given the minimal influence of material type on overall contamination, these findings suggest that environmental control measures may be more effective than material selection alone in managing bioburden.

Future applications should integrate molecular tools with environmental monitoring to achieve more comprehensive characterization of surface contamination. Approaches such as ITS metabarcoding can reveal the total fungal community, including slow-growing or nonculturable taxa, thereby complementing culture-based enumeration. Combining these molecular insights with continuous tracking of environmental parameters would provide both taxonomic resolution and predictive capacity. Together, these strategies offer industries a practical pathway to reduce material waste, limit biodeterioration of products, and strengthen overall contamination control in manufacturing environments.

## Supplementary Material

qvaf029_Supplemental_Files

## Data Availability

The data underlying this article will be shared on reasonable request to the corresponding author.
