# Supplementary material for: Fungal contamination patterns and surface material susceptibility in manufacturing environments
Source: Sustain Microbiol. 2025 Dec 3;2(4):qvaf029. doi: 10.1093/sumbio/qvaf029 (PMC13375379; doi:10.1093/sumbio/qvaf029)
Supplement: Supplementary file 1 [file qvaf029_Supplemental_Files.zip › Table S1. List of surface materials sampled for this study.docx]

Table S1. List of surface materials sampled for this study.

| Surface | Materials |
| --- | --- |
| Wood | Wooden trolley |
|  | Wooden pallet |
|  | Wooden racks |
|  | Wooden table |
| Fabric | Synthetic fabric |
|  | Finished footwear |
|  | Thread |
|  | Meshed fabric |
|  | Cleaning fabric |
| Leather | Synthetic leather on shoes |
|  | Suede |
|  | Leather cover |
|  | Raw synthetic leather |
| Plastic | Polyurethane conveyor belt |
|  | Stockfitting mold |
|  | Plastic covered tables |
|  | Plastic curtain |
|  | Plastic basket |
|  | Protective plastic cover |
| Rubber | Shoes outsoles |
|  | Shoes midsoles |
|  | Rubber strip curtains |
|  | Rubber tables |
