# Supplementary material for: Fungal contamination patterns and surface material susceptibility in manufacturing environments
Source: Sustain Microbiol. 2025 Dec 3;2(4):qvaf029. doi: 10.1093/sumbio/qvaf029 (PMC13375379; doi:10.1093/sumbio/qvaf029)
Supplement: Supplementary file 1 [file qvaf029_Supplemental_Files.zip › Table S2. Sequenced sample Match with Genbank.docx]

Table S2. Sequenced sample Match with Genbank.

| No. | Sample name | Species | Accession | %match |
| --- | --- | --- | --- | --- |
| 1 | 001_A01_B250103M1C | *Alternaria* sp. | [PV597461.1](https://www.ncbi.nlm.nih.gov/nucleotide/PV597461.1?report=genbank&log$=nuclalign&blast_rank=1&RID=CS3J45ER014) | 99% |
| 2 | 008_H01_B25010303A | *Annulohypoxylon* sp. | [OR900106.1](https://www.ncbi.nlm.nih.gov/nucleotide/OR900106.1?report=genbank&log$=nuclalign&blast_rank=1&RID=CS246NNS014) | 99.43% |
| 3 | 006_F01_A25010201C | *Apiospora* sp. | [PV597259.1](https://www.ncbi.nlm.nih.gov/nucleotide/PV597259.1?report=genbank&log$=nuclalign&blast_rank=1&RID=CS3J9PPP015) | 100% |
| 4 | 059_C08_B250103M1A | *Aplosporella hesperidica* | [JX681069.1](https://www.ncbi.nlm.nih.gov/nucleotide/JX681069.1?report=genbank&log$=nuclalign&blast_rank=1&RID=CS6M522R015) | 99.83% |
| 5 | 062_F08_B25010317A | *Ascotricha sinuosa* | [MK805508.1](https://www.ncbi.nlm.nih.gov/nucleotide/MK805508.1?report=genbank&log$=nuclalign&blast_rank=1&RID=CS50HY17015) | 99.64% |
| 6 | 089_A12_C24110501B | *Aspergillus aculeatus* | [OL614445.1](https://www.ncbi.nlm.nih.gov/nucleotide/OL614445.1?report=genbank&log$=nuclalign&blast_rank=4&RID=CS5R7E2E015) | 99.64% |
| 7 | 010_B02_B25010307C | *Aspergillus foetidus* | [OM436872.1](https://www.ncbi.nlm.nih.gov/nucleotide/OM436872.1?report=genbank&log$=nuclalign&blast_rank=1&RID=CS3EHBSY014) | 99.49% |
| 8 | 089_A12_C24110513A | *Aspergillus fumigatus* | [ON307213.1](https://www.ncbi.nlm.nih.gov/nucleotide/ON307213.1?report=genbank&log$=nuclalign&blast_rank=1&RID=CS5RF3JE015) | 99.65% |
| 9 | 096_H12_C24110528B | *Aspergillus fumigatus* | [MT141152.1](https://www.ncbi.nlm.nih.gov/nucleotide/MT141152.1?report=genbank&log$=nuclalign&blast_rank=2&RID=CS6M4MZF015) | 99.83% |
| 10 | 091_C12_C24110513C | *Aspergillus fumigatus* | [KF494830.1](https://www.ncbi.nlm.nih.gov/nucleotide/KF494830.1?report=genbank&log$=nuclalign&blast_rank=1&RID=CS6BVWHN014) | 99.65% |
| 11 | 013_E02_B25010312D | *Aspergillus fumigatus* | [PP781243.1](https://www.ncbi.nlm.nih.gov/nucleotide/PP781243.1?report=genbank&log$=nuclalign&blast_rank=1&RID=CS3SWM7A015) | 99% |
| 12 | 081_A11_C24110527A | *Aspergillus* sp. | [PQ851033.1](https://www.ncbi.nlm.nih.gov/nucleotide/PQ851033.1?report=genbank&log$=nuclalign&blast_rank=1&RID=CS581VXE014) | 99.49% |
| 13 | 015_G02_B25010322A | *Aspergillus* sp. | [PV291760.1](https://www.ncbi.nlm.nih.gov/nucleotide/PV291760.1?report=genbank&log$=nuclalign&blast_rank=1&RID=CS41T7M0014) | 99.46% |
| 14 | 076_D10_C24110501A | *Aspergillus* sp. | [MK108386.1](https://www.ncbi.nlm.nih.gov/nucleotide/MK108386.1?report=genbank&log$=nuclalign&blast_rank=1&RID=CS50ZAS8015) | 99.66% |
| 15 | 095_G12_C24110518A | *Aspergillus* sp. | [PX088178.1](https://www.ncbi.nlm.nih.gov/nucleotide/PX088178.1?report=genbank&log$=nuclalign&blast_rank=1&RID=CS6HYSYG014) | 99.29% |
| 16 | 084_D11_C24110530B | *Aureobasidium pullulans* | [MZ501697.1](https://www.ncbi.nlm.nih.gov/nucleotide/MZ501697.1?report=genbank&log$=nuclalign&blast_rank=2&RID=CS588H3S015) | 99.30% |
| 17 | 057_A08_A25010201B | *Cladosporium* sp. | [OR351476.1](https://www.ncbi.nlm.nih.gov/nucleotide/OR351476.1?report=genbank&log$=nuclalign&blast_rank=1&RID=CS4RVYJB014) | 99.81% |
| 18 | 015_G02_B25010303B | *Coprinellus radians* | [MZ621141.1](https://www.ncbi.nlm.nih.gov/nucleotide/MZ621141.1?report=genbank&log$=nuclalign&blast_rank=2&RID=CS41H2ZT015) | 99.54% |
| 19 | 094_F12_C24110516A | *Coprinellus radians* | [MN622748.1](https://www.ncbi.nlm.nih.gov/nucleotide/MN622748.1?report=genbank&log$=nuclalign&blast_rank=1&RID=CS6HSZX8015) | 99.10% |
| 20 | 013_E02_B25010302A | *Coprinellus* sp. | [PQ726996.1](https://www.ncbi.nlm.nih.gov/nucleotide/PQ726996.1?report=genbank&log$=nuclalign&blast_rank=1&RID=CS3SM8T0014) | 99.40% |
| 21 | 012_D02_B25010311A | *Coprinopsis cinerea* | [KP724995.1](https://www.ncbi.nlm.nih.gov/nucleotide/KP724995.1?report=genbank&log$=nuclalign&blast_rank=1&RID=CS3SCSCC015) | 99.70% |
| 22 | 093_E12_C24110513D | *Curvularia pseudobrachyspora* | [PX048752.1](https://www.ncbi.nlm.nih.gov/nucleotide/PX048752.1?report=genbank&log$=nuclalign&blast_rank=1&RID=CS4GU4K9015) | 99.82% |
| 23 | 008_H01_A25010203G | *Curvularia* sp. | [OQ793696.1](https://www.ncbi.nlm.nih.gov/nucleotide/OQ793696.1?report=genbank&log$=nuclalign&blast_rank=2&RID=CS1ZSB69015) | 99.82% |
| 24 | 014_F02_B25010313B | *Dirkmeia* sp. | [MN515015.1](https://www.ncbi.nlm.nih.gov/nucleotide/MN515015.1?report=genbank&log$=nuclalign&blast_rank=2&RID=CS41AE2X015) | 99.59% |
| 25 | 026_B04_B25010318B | *Fusarium solani* | [PP581789.1](https://www.ncbi.nlm.nih.gov/nucleotide/PP581789.1?report=genbank&log$=nuclalign&blast_rank=2&RID=CS4RNPH2015) | 99.26% |
| 26 | 078_F10_C24110501D | *Fusarium* sp. | [MH884157.1](https://www.ncbi.nlm.nih.gov/nucleotide/MH884157.1?report=genbank&log$=nuclalign&blast_rank=1&RID=CS5792D5015) | 99.26% |
| 27 | 022_F03_B25010314A | *Fusarium verticillioides* | [PQ579861.1](https://www.ncbi.nlm.nih.gov/nucleotide/PQ579861.1?report=genbank&log$=nuclalign&blast_rank=1&RID=CS4GKT7H015) | 99.63% |
| 28 | 079_G10_C24110501E | *Neurospora crassa* | [KR085965.1](https://www.ncbi.nlm.nih.gov/nucleotide/KR085965.1?report=genbank&log$=nuclalign&blast_rank=1&RID=CS57MT0F015) | 99.31% |
| 29 | 089_A12_C241105M6C | *Nigrospora oryzae* | [MZ148528.1](https://www.ncbi.nlm.nih.gov/nucleotide/MZ148528.1?report=genbank&log$=nuclalign&blast_rank=3&RID=CS5R07BH015) | 100.00% |
| 30 | 020_D03_C24110527B | *Nigrospora oryzae* | [OR984156.1](https://www.ncbi.nlm.nih.gov/nucleotide/OR984156.1?report=genbank&log$=nuclalign&blast_rank=1&RID=CS4G1KCV015) | 99.63% |
| 31 | 088_H11_C24110511A | *Nigrospora* sp. | [KJ863496.1](https://www.ncbi.nlm.nih.gov/nucleotide/KJ863496.1?report=genbank&log$=nuclalign&blast_rank=1&RID=CS5H82C4015) | 99.63% |
| 32 | 086_F11_C241105M3A | *Nigrospora* sp. | [KJ863500.1](https://www.ncbi.nlm.nih.gov/nucleotide/KJ863500.1?report=genbank&log$=nuclalign&blast_rank=1&RID=CS5GH3WA015) | 99.63% |
| 33 | 087_G11_C241105M5E | *Nigrospora sphaerica* | [MN121395.1](https://www.ncbi.nlm.nih.gov/nucleotide/MN121395.1?report=genbank&log$=nuclalign&blast_rank=1&RID=CS5GUDAW015) | 100% |
| 34 | 007_G01_B250103M1E | *Nigrospora sphaerica* | [KU196743.1](https://www.ncbi.nlm.nih.gov/nucleotide/KU196743.1?report=genbank&log$=nucltop&blast_rank=1&RID=CS1X2354014) | 99.63% |
| 35 | 077_E10_C24110501C | *Penicillium citrinum* | [MH591452.1](https://www.ncbi.nlm.nih.gov/nucleotide/MH591452.1?report=genbank&log$=nuclalign&blast_rank=1&RID=CS517N44015) | 100% |
| 36 | 002_B01_B25010312A | *Phaeosphaeriopsis* sp. | [JQ936185.1](https://www.ncbi.nlm.nih.gov/nucleotide/JQ936185.1?report=genbank&log$=nuclalign&blast_rank=1&RID=CS3J69GK015) | 99.44% |
| 37 | 085_E11_C24110531B | *Phlebiopsis gigantea* | [OP315779.1](https://www.ncbi.nlm.nih.gov/nucleotide/OP315779.1?report=genbank&log$=nuclalign&blast_rank=1&RID=CS5GB3PW014) | 98.76% |
| 38 | 014_F02_B25010302B | *Porostereum fulvum* | [PP582951.1](https://www.ncbi.nlm.nih.gov/nucleotide/PP582951.1?report=genbank&log$=nuclalign&blast_rank=1&RID=CS3T05CC015) | 98.54% |
| 39 | 088_H11_C241105M6B | *Sarocladium oryzae* | [MK902763.1](https://www.ncbi.nlm.nih.gov/nucleotide/MK902763.1?report=genbank&log$=nuclalign&blast_rank=1&RID=CS5H13NZ015) | 99.65% |
| 40 | 020_D03_B25010310B | *Trichoderma longibrachiatum* | [JN108926.1](https://www.ncbi.nlm.nih.gov/nucleotide/JN108926.1?report=genbank&log$=nuclalign&blast_rank=1&RID=CS4GAXGN015) | 99.22% |
